# The role of CARMA3 in regulating fibrosis to prevent hypertrophic cardiomyopathy

**DOI:** 10.1038/s41420-025-02645-z

**Published:** 2025-10-06

**Authors:** Yafeng Liu, Ganyi Chen, Yiwei Yao, Yunfei Jiang, Chenghao Wen, Wuwei Wang, Quan Liu, Yide Cao, Fuhua Huang, Wen Chen, Zhibing Qiu

**Affiliations:** 1 Department of Thoracic and Cardiovascular Surgery, Nanjing First Hospital, Nanjing Medical University, Nanjing, Jiangsu China; 2 Department of Thoracic and Cardiovascular Surgery, Nanjing First Hospital, Southeast University School of Medicine, Nanjing, Jiangsu China

**Keywords:** Cell biology, Cell growth

## Abstract

Hypertrophic cardiomyopathy (HCM) is characterized by cardiac hypertrophy and fibrosis. To investigate the impact of CARMA3 on fibroblast phenotypic transformation in hypertrophic cardiomyopathy (HCM), the correlation between CARMA3 expression and fibrosis was analyzed in HCM patients. Cardiac function and fibroblast phenotypic transformation were assessed in wild-type and CARMA3-knockout mice subjected to transverse aortic constriction (TAC) or angiotensin II treatment. Additionally, cardiac fibroblasts were screened using flow cytometry and proteomic analysis to identify potential targets. Significant cardiac functional impairment and fibrosis were observed in CARMA3-knockout mice following TAC or angiotensin II treatment. Primary fibroblasts isolated from these mice exhibited increased myofibroblast differentiation, extracellular collagen production, mitochondrial damage, and macrophage inflammation. Elevated STAT1 expression was identified in cardiac fibroblasts from CARMA3-knockout mice through proteomic analysis. Additionally, STAT1 phosphorylation was regulated by CARMA3, and an interaction between CARMA3 and STAT1 was detected in response to pressure overload. In conclusion, CARMA3 may suppress myofibroblast activation by inhibiting STAT1 phosphorylation, thereby improving myocardial fibrosis in pressure overload-induced HCM.

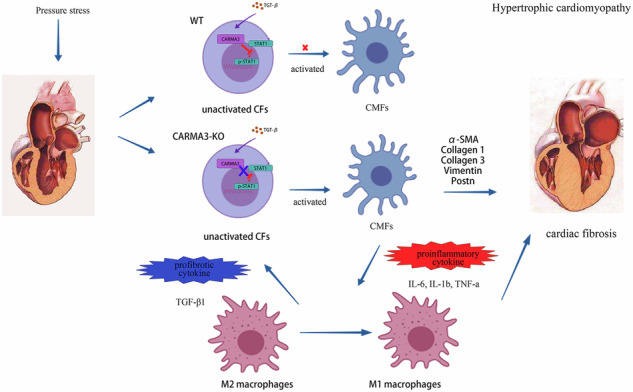

## Introduction

With the aging global population, the incidence of hypertrophic cardiomyopathy (HCM) is on the rise [1]. Cardiac hypertrophy, a hallmark of HCM, is often accompanied by adverse remodeling, including cardiomyocyte hypertrophy and fibrosis [2, 3]. Although initially considered an adaptive response, chronic cardiac hypertrophy can ultimately lead to impaired cardiac function and heart failure. Therefore, understanding the molecular mechanisms underlying cardiac hypertrophy [3] and identifying novel therapeutic targets are essential for improving patient management. Previous research on cardiac hypertrophy has primarily focused on cardiomyocytes [4–6]. Nonetheless, myocardial fibrosis, driven by cardiac fibroblasts (CFs), also plays a crucial role in adverse cardiac remodeling [7, 8]. In response to harmful stimuli, CFs produce excessive extracellular matrix (ECM), leading to fibrotic scar formation that compromises cardiac function [9].

Emerging evidence indicates that CFs are essential for maintaining adjacent cell homeostasis and are involved in various heart diseases [10]. They secrete cytokines, adhesion molecules, and growth factors that influence the function of cardiomyocytes, immune cells, and other cell types [11–13]. Moreover, CFs contribute to ECM homeostasis [10], providing structural support for cardiomyocytes and regulating the heart’s biochemical, molecular, and structural properties [14]. In response to cardiac tissue injury or stimulation, CFs activate, leading to proliferation, migration to the injury site, ECM deposition, and cardiac remodeling [15]. Importantly, excessive CF activation and proliferation following myocardial injury can contribute to adverse cardiac remodeling and chronic heart failure [16, 17]. During this process, CFs transition from a pro-inflammatory phenotype to an anti-inflammatory, pro-fibrotic cardiac myofibroblast (CMF) phenotype [18, 19]. Thus, deciphering the mechanisms underlying abnormal CF activation and cardiac fibrosis is paramount for preventing cardiac hypertrophy and dysfunction. However, the regulatory processes governing CF phenotypic transitions remain incompletely understood.

Caspase recruitment domain and membrane-associated guanylate kinase-like protein 3 (CARMA3) is a member of the CARMA family, widely expressed in various tissues. Previous studies have shown that CARMA3 can form the CARMA-BCL10-MALT1 complex, which is involved in the development of several diseases [20]. The CARMA3-BCL10-MALT1 signalosome mediates angiotensin II (Ang II) type 1 receptor signaling to modulate vascular inflammation and atherosclerosis [21]. Besides, recent research has demonstrated that CARMA3 can modulate ECM expression [22]. Similarly, our previous studies have highlighted the involvement of CARMA3 in cardiovascular diseases and pulmonary fibrosis [23, 24]. However, the role of CARMA3 in cardiac hypertrophy and fibrosis remains elusive. Therefore, the present study aimed to investigate the biological functions and underlying mechanisms of CARMA3 in these pathological processes.

## Results

### CARMA3 was decreased with myocardial fibrosis development

**Fig. 1:**
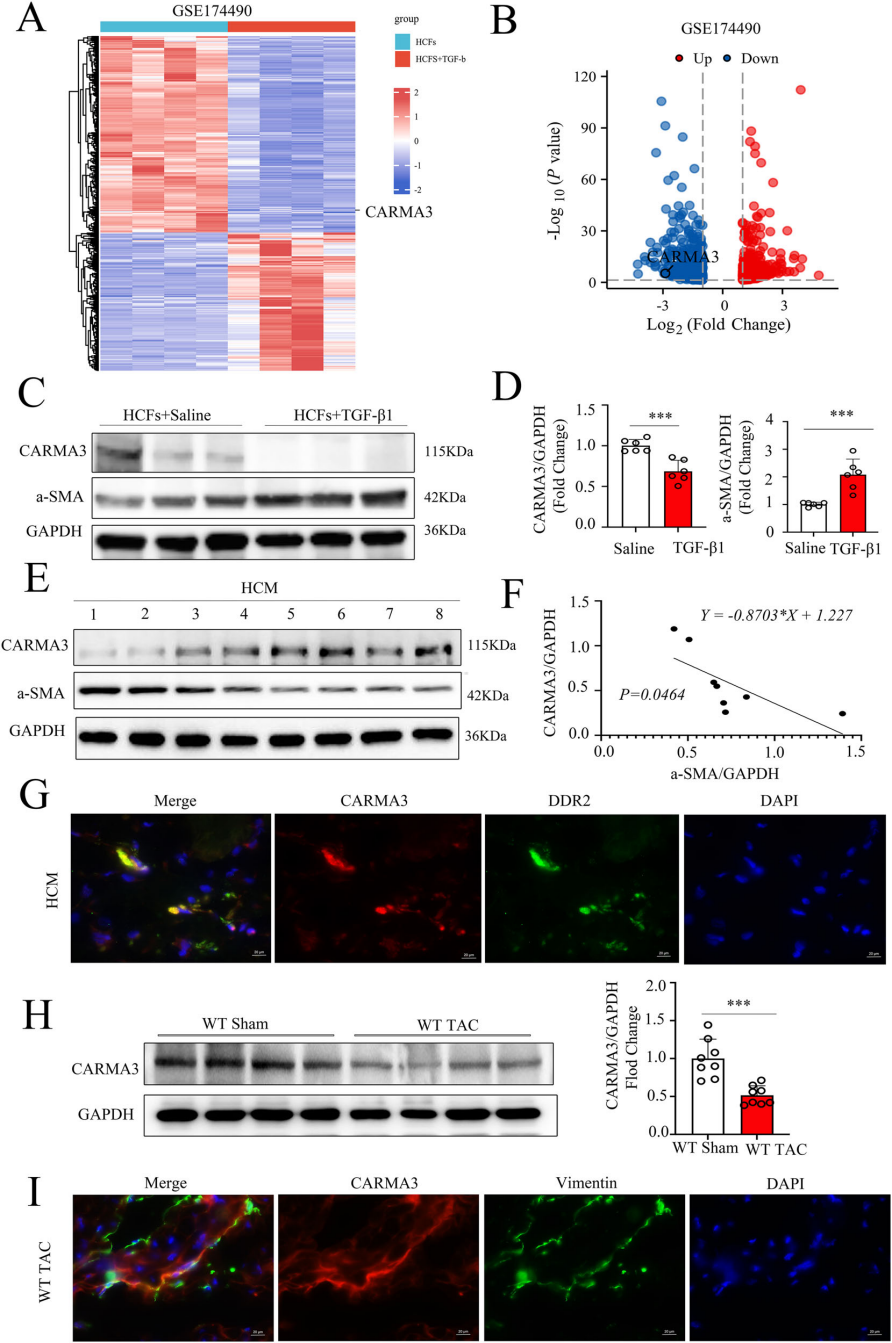
CARMA3 was decreased with myocardial fibrosis development. **A** Heat map of differential genes in GSE174490. **B** Volcano plot of differential genes in GSE174490 *n* = 4, per group. **C**, **D** Representative Western Blot of CARMA3 and α-SMA in primary fibroblasts isolated from the human heart with TGF-β1 stimulation were performed with GAPDH as a loading control, *n* = 6, per group. **E** Representative Western Blot of CARMA3 and α-SMA in heart of patients with hypertrophic cardiomyopathy was performed with GAPDH as a loading control, *n* = 8. **F** Statistical plot of linear correlation between CARMA3 and α-SMA in cardiac tissue from patients with hypertrophic cardiomyopathy. **G** Representative immunostaining images for CARMA3 (red) and DDR2 (green) in the heart of patients with hypertrophic cardiomyopathy, *n* = 6, per group. Magnification 1000×. **H** Representative Western Blot of CARMA3 in the heart of wild-type mice after TAC or Sham, *n* = 8, per group. **I** Representative immunostaining images for CARMA3 (red) and Vimentin (green) in the heart of wild-type mice after TAC. Magnification 1000×, *n* = 6, per group. *P < 0.05, ***P < 0.001.

To identify differentially expressed genes (DEGs) associated with fibroblast phenotypic transformation, analysis of the public dataset GSE174490 revealed a significant reduction in CARMA3 expression in activated myofibroblasts (Fig. 1A, B). To validate these findings, primary human cardiac fibroblasts were isolated, cultured, and stimulated with TGF-β 1. TGF-β 1 treatment resulted in a significant increase in α-SMA protein levels and a decrease in CARMA3 protein levels (Fig. 1C, D). Next, hypertrophic myocardial tissues were collected from eight patients who underwent Morrow myectomy (Table S1) to further investigate the relationship between CARMA3 expression and fibrosis. Interestingly, our results revealed that CARMA3 protein levels decreased with increasing fibrosis severity(Fig. 1E, F). Meanwhile, immunofluorescence analysis revealed co-localization of CARMA3 with the fibroblast marker protein DDR2 in myocardial tissues from patients with hypertrophic myocardium (Fig. 1G). Furthermore, a significant reduction in CARMA3 expression (Fig. 1H) was observed, with notable CARMA3 co-localization with the fibroblast marker protein, Vimentin, (Fig. 1I) in mice with hypertrophic cardiomyopathy. The above findings suggest that CARMA3 plays an essential regulatory role in hypertrophic cardiomyopathy.

### CARMA3 deficiency aggravates cardiac dysfunction and adverse ventricular remodeling by pressure overload

**Fig. 2:**
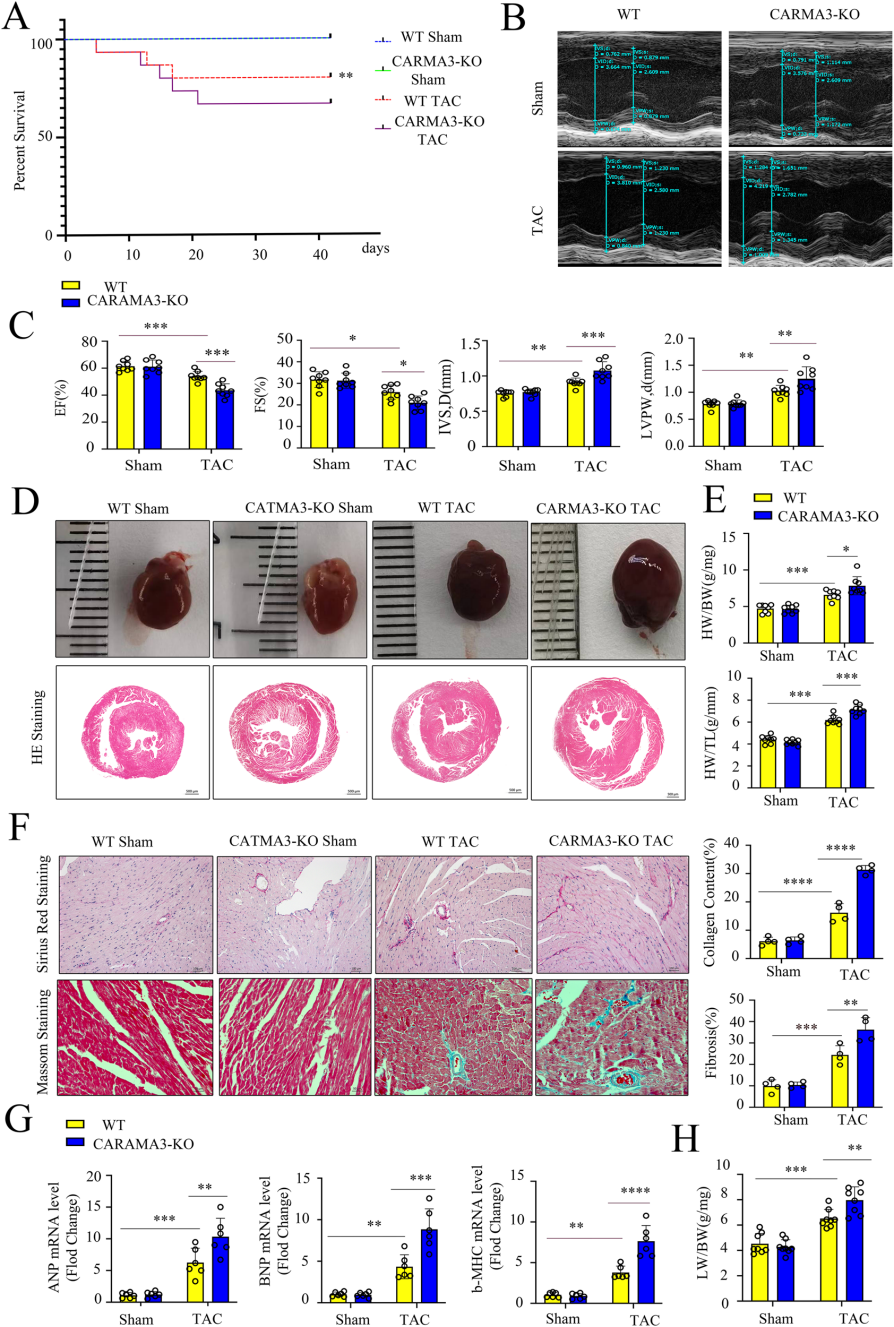
CARMA3 deficiency aggravates cardiac dysfunction and adverse ventricular remodeling by pressure overload. **A** Changes in mice survival in wild-type and CARMA3 KO mice after TAC or Sham. **B**, **C**. Echocardiography of wild-type and CARMA3 KO mice heart and EF, FS, IVSD and LVPW. **D** Images of whole mice heart and HE staining. **E** Quantification of the heart weight/body weight ratio and the heart weight/tibial length ratio. **F** Images of Masson (C) and picrosirius red staining(Magnification 200×) of mice heart and quantification of fibrotic area. **G** mRNA levels of ANP, BNP, and β-MHC in mice heart after TAC. **H** Quantification of the wet lung weight/body weight ratio. *n* = 6, per group, *P < 0.05, **P < 0.01, ***P < 0.001, ****P < 0.0001.

To investigate the role of CARMA3 in cardiac function during TAC-induced cardiac hypertrophy, we performed a 6-week in vivo TAC procedure. We found that mortality was higher in CARMA3 knockout (KO) mice compared to wild-type (WT) mice (Fig. 2A). Echocardiographic analysis revealed more marked increases in interventricular septal thickness (IVSD) and left ventricular posterior wall thickness (LVPW), along with more significant decreases in left ventricular ejection fraction (LVEF) and fractional shortening (FS) in CARMA3 KO mice (Fig. 2B, C). Histological analysis, including hematoxylin and eosin (H&E) staining, heart weight-to-body weight ratio, and heart weight-to-tibial length ratio, revealed more severe cardiomyocyte hypertrophy in CARMA3 KO mice (Fig. 2D, E). Masson’s trichrome and Sirius Red staining showed increased collagen deposition and fibrosis in CARMA3 KO mice (Fig. 2F). Besides, mRNA levels of heart failure markers (ANP, BNP, and β-MHC) were significantly elevated in CARMA3 KO mice (Fig. 2G, H). The increased wet lung weight-to-body weight ratio further confirmed more severe heart failure in CARMA3 KO mice (Fig. 2H). Collectively, these findings demonstrate that CARMA3 deficiency exacerbates cardiac dysfunction, adverse ventricular remodeling, and heart failure under pressure overload.

To further elucidate the role of CARMA3 in hypertrophic cardiomyopathy, mice were treated with Ang II for 4 weeks. CARMA3 KO mice exhibited significantly higher mortality (Fig. S1A), increased IVSD, LVPW, and left ventricular diastolic internal diameter (LVDI) (Fig. S1B), as well as an elevated heart weight-to-body weight ratio (Fig. S1D, E). Moreover, CARMA3 KO mice showed enhanced collagen deposition and fibrosis (Fig. S1F), increased expression levels of heart failure markers (ANP, BNP, and β-MHC), and a higher wet lung weight-to-body weight ratio (Fig. S1G, H). Furthermore, CARMA3 KO mice displayed substantially lower LVEF and fractional shortening (FS) (Fig. S1B). These results suggest that CARMA3 deficiency also exacerbates cardiac dysfunction in Ang II-induced hypertrophic cardiomyopathy.

### CARMA3 deficiency promotes myofibroblast activation under pressure overload

**Fig. 3:**
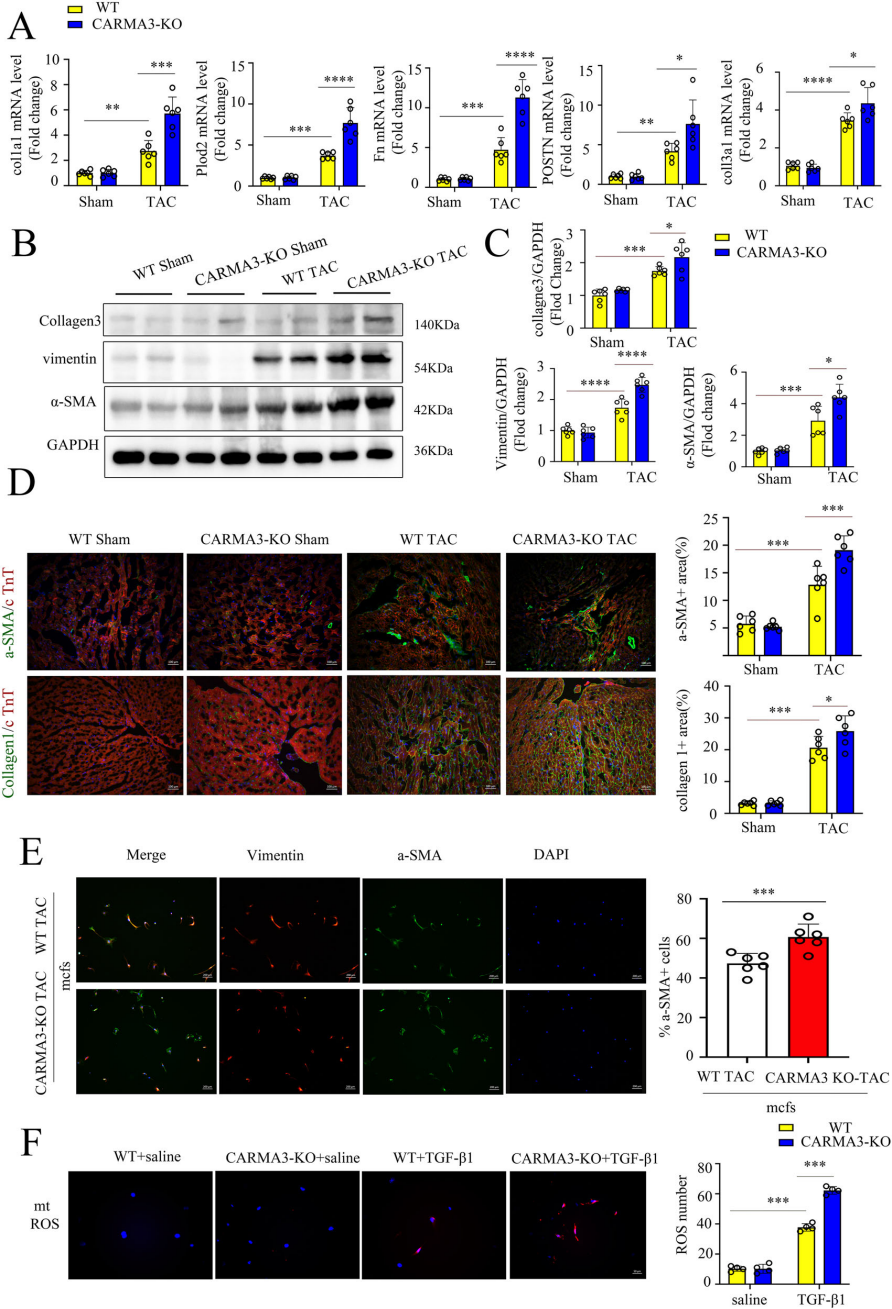
CARMA3 deficiency promotes myofibroblast activation under pressure overload. **A** mRNA levels of Col1a1, Col3a1, Fn, POSTN and Plod2 of mice heart after TAC or Sham, n = 6, per group. **B**, **C** Western Blot of collagen3, vimentin and α-SMA in mice heart after TAC was performed with GAPDH as a loading control, *n* = 6, per group. **D** Representative immunostaining images for α-SMA (green), collagen1 (green), and cTNT (red) in the heart after TAC. Magnification 200×, *n* = 6, per group. **E** Representative immunostaining images for α-SMA (green) and vimentin (red) in primary fibroblast from mice heart after TAC. Magnification 100×, *n* = 6, per group. **F** Representative immunostaining images for mtROS (red) in primary fibroblast with TGF- β1. Magnification 400×. *n* = 4, per group, *P < 0.05, **P < 0.01, ***P < 0.001, ****P < 0.0001.

To elucidate the role of CARMA3 in fibroblast phenotypic transformation, we examined the mRNA levels of collagen-secreting protein markers (Col1a1 and Col3a1) and extracellular matrix proteins (Fn, POSTN, and Plod2) in cardiac tissues. Our findings revealed that CARMA3 deficiency significantly increased the mRNA expression levels of these phenotypic transformation markers (Fig. 3A). In addition, CARMA3 KO mice exhibited increased protein levels of the cardiac myofibroblast CMF marker α-SMA and extracellular collagen-secreting proteins (Collagen3 and Vimentin) following six weeks of TAC surgery (Fig. 3B, C). Immunofluorescence co-staining further demonstrated elevated protein expression of α-SMA and Collagen1 in the myocardium of CARMA3 KO mice (Fig. 3D), with a significant increase in α-SMA-positive CMFs observed in primary fibroblasts isolated from the hearts of CARMA3 KO mice (Fig. 3E). Additionally, we investigated the impact of CARMA3 on fibroblast mitochondrial function. Our results indicated that CARMA3 deficiency exacerbated mitochondrial dysfunction, leading to increased production of mitochondrial reactive oxygen species (mtROS) and impaired mitochondrial membrane potential (Fig. 3F and Fig. S2A). Collectively, these findings suggest that CARMA3 deficiency enhances fibroblast phenotypic transformation and activation under pressure overload.

Western blot analysis showed that CARMA3 deficiency further increased the expression of α-SMA and extracellular collagen-secreting proteins, including Collagen3 and Vimentin, in cardiac tissues following Ang II stimulation (Fig. S3A). Besides, immunohistochemical staining demonstrated elevated levels of POSTN and Collagen1 in the cardiac tissues of CARMA3 knockout mice subjected to Ang II treatment (Fig. S3B).

### CARMA3 deficiency increases the expression of proinflammatory macrophages

**Fig. 4:**
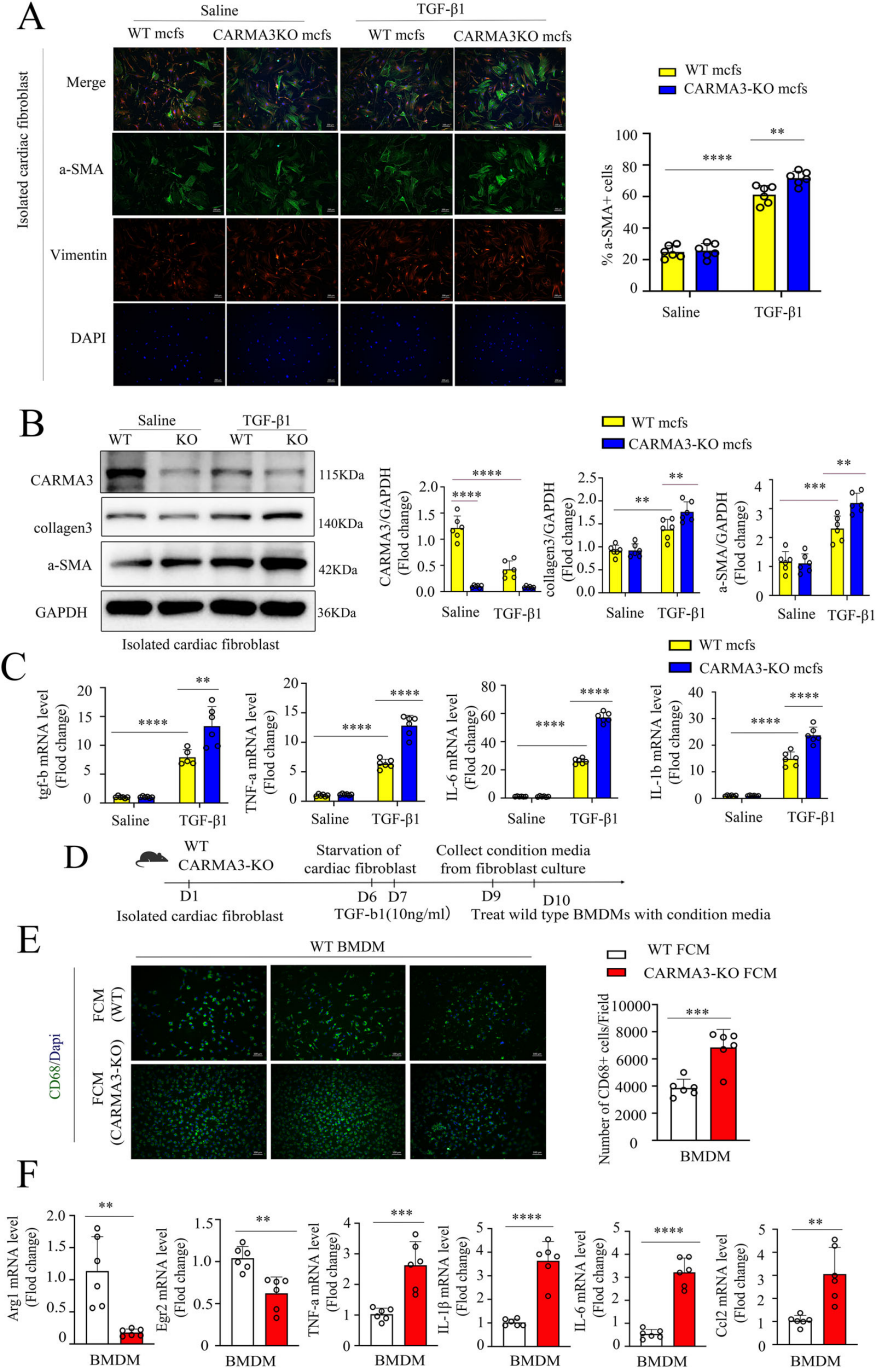
CARMA3 deficiency increases the expression of proinflammatory macrophages. **A** Representative immunostaining images for α-SMA (green) and vimentin (red) in primary fibroblast from mice heart with TGF-β1 stimulation. Magnification 100×. **B** Western Blot of collagen3, vimentin and α-SMA in primary fibroblast from mice heart with TGF-β1 stimulation was performed with GAPDH as a loading control. **C** mRNA levels of TGF-β, TNF-α, IL-1β and IL-6 in primary fibroblast from mice heart with TGF-β1 stimulation. **D** Flow chart of fibroblast and macrophage co-culture. **E** Representative immunostaining images for CD68 (green) in macrophages co-cultured with fibroblast. Magnification 200×. **F** mRNA levels of Arg1, Erg2, TNF-α, IL-1β, IL-6, and CCL2 in macrophages co-cultured with fibroblast. *n* = 6, per group, **P < 0.01, ***P < 0.001, ****P < 0.0001.

Next, primary CFs were isolated from WT and CARMA3 KO mice and stimulated with TGF-β1. Immunofluorescence double staining revealed a significant increase in the proportion of α-SMA-positive cells in CARMA3 KO CFs under TGF-β1 stimulation (Fig. 4A). Moreover, CARMA3 KO CFs exhibited higher expression levels of α-SMA and the extracellular collagen marker Collagen3 (Fig. 4B). The expression of TGF-β and proinflammatory factors, including TNF-α, IL-1β, and IL-6, was also significantly elevated in CARMA3 KO CFs following TGF-β1 stimulation (Fig. 4C). To further explore the impact of CFs on macrophage function, we conducted a co-culture experiment with macrophages (Fig. 4D). To investigate the effect of the cardiac fibroblast secretome on macrophage migration and polarization, wild-type BMDMs were treated with a conditioned medium collected from cultured cardiac fibroblasts. The BMDM migration assay demonstrated that conditioned medium from CARMA3 KO fibroblasts significantly enhanced BMDM migration, suggesting that CARMA3 deficiency in fibroblasts accelerates macrophage recruitment (Fig. 4E). Furthermore, quantitative PCR analysis demonstrated a significant decrease in Arg1 and Erg2 expression, along with a notable increase in TNF-α, IL-1β, IL-6, and CCL2 expression in macrophages co-cultured with CARMA3 KO CFs (Fig. 4F). These results suggest that CARMA3 deficiency enhances the polarization of macrophages toward a pro-inflammatory phenotype.

### STAT1 signaling plays a key role in hypertrophic cardiomyopathy

**Fig. 5:**
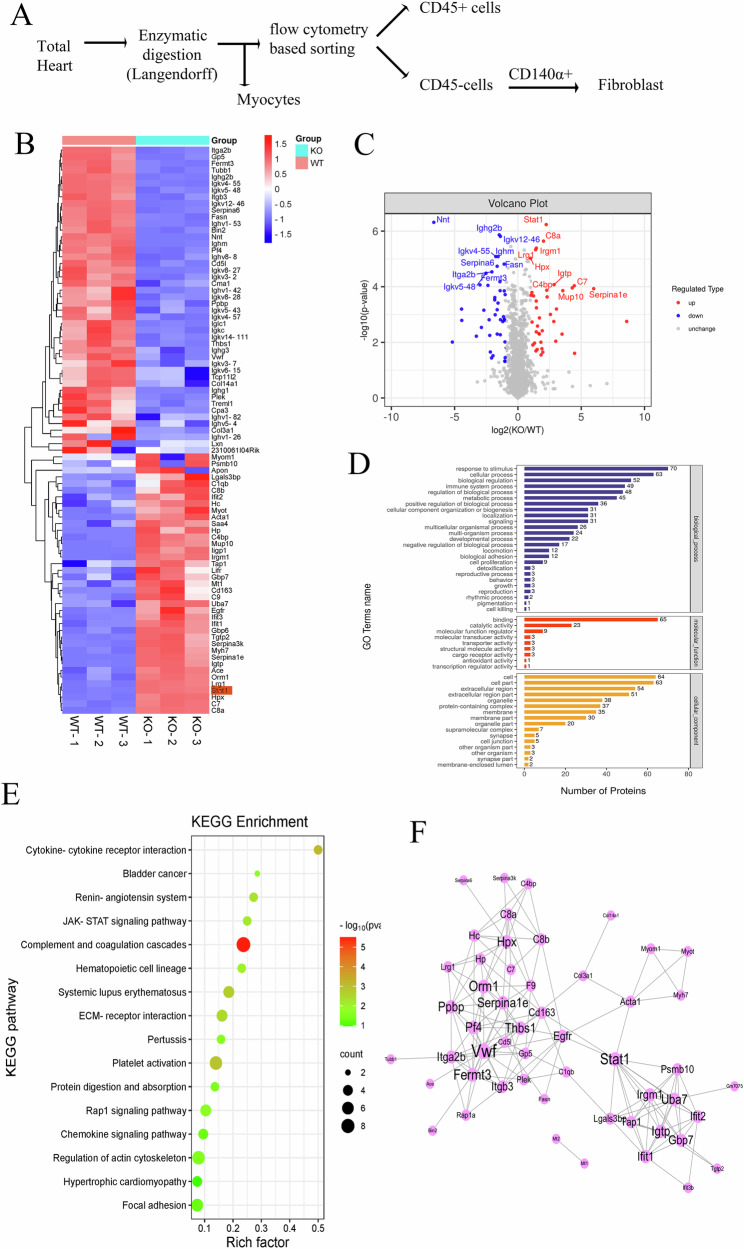
STAT1 signaling plays a key role in hypertrophic cardiomyopathy. **A** Flow chart of sorting cells. **B** Heat map of differential genes in primary fibroblast from wild-type and CARMA3 KO mice heart after TAC. **C** Volcano plot of differential genes in primary fibroblast from wild-type and CARMA3 KO mice heart after TAC. **D** GO analysis of differential genes in primary fibroblast from wild-type and CARMA3 KO mice heart after TAC including biological process (blue), molecular function (red) and cellular component (orange). **E** KEGG analysis of differential genes in primary fibroblast from wild-type and CARMA3 KO mice heart after TAC. **F** Diagram of the interaction network of differential proteins.

CFs from WT and CARMA3 KO mice were analyzed using flow cytometry following TAC surgery (Fig. 5A). Four-dimensional label-free proteomics technology identified 39 significantly upregulated and 46 significantly DEGs (Fig. 5B). Notably, STAT1 expression was markedly higher in CARMA3 KO mice (Fig. 5B, C). Gene Ontology (GO) analysis showed that these differentially expressed genes were primarily associated with biological regulation, immune system regulation (biological processes), catalytic activity, and signal transduction (molecular functions) (Fig. 5D). Kyoto Encyclopedia of Genes and Genomes (KEGG) analysis highlighted their involvement in hypertrophic cardiomyopathy, the renin-angiotensin system, and JAK-STAT signaling (Fig. 5E). Protein interaction network analysis further highlighted the key regulatory role of STAT1 (Fig. 5F). These results suggest that CARMA3 might regulate fibroblast behavior in pressure overload-induced hypertrophic cardiomyopathy by targeting STAT1.

### CARMA3 deficiency activates STAT1 signaling

**Fig. 6:**
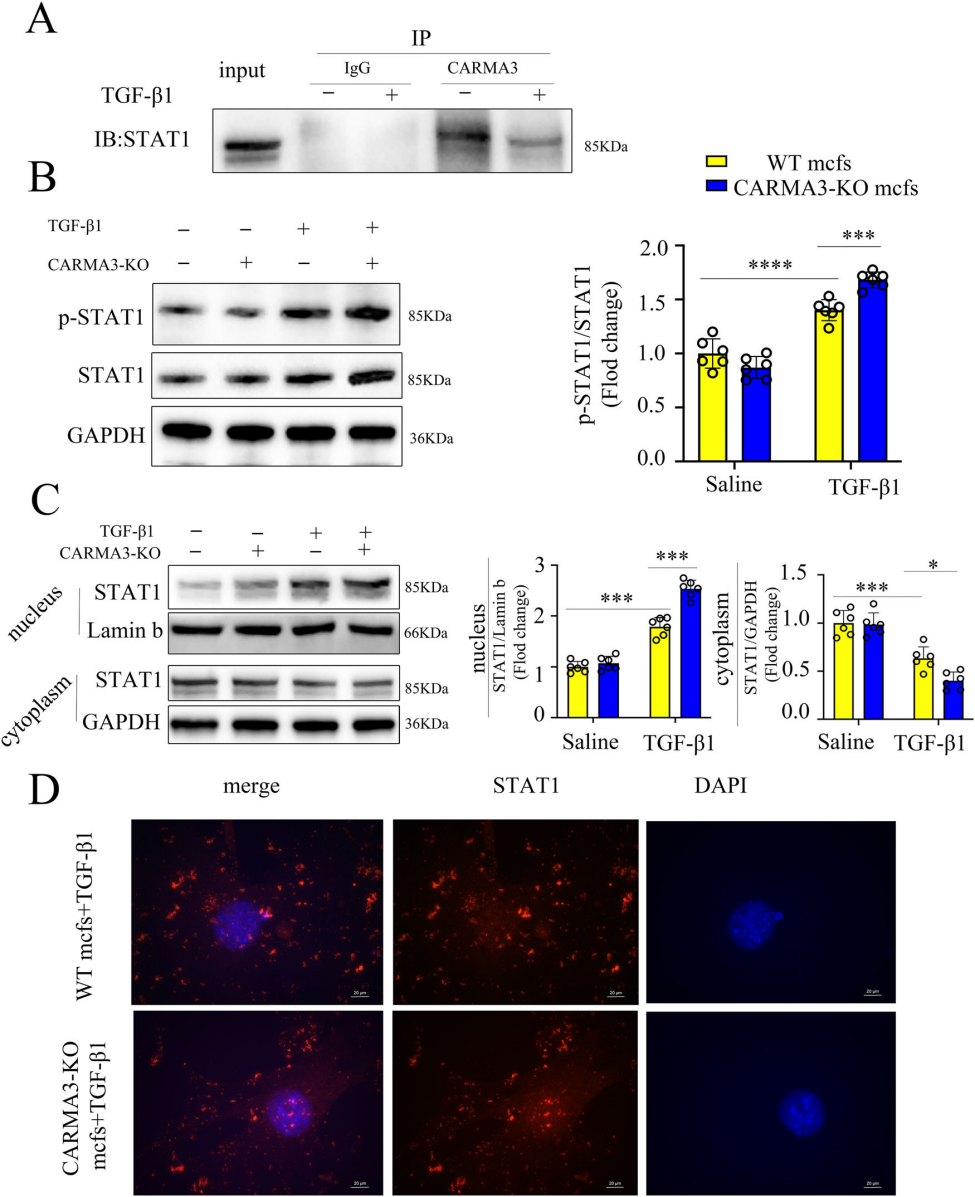
CARMA3 deficiency activates STAT1 signaling. **A** CO-IP analysis of CARMA3 and STAT1 in primary fibroblast from mice heart with TGF-β1 stimulation. **B** Western Blot of p-STAT1 and STAT1 in primary fibroblast from mice heart with TGF-β1 stimulation was performed with GAPDH as a loading control. **C** Western Blot of STAT1 in the nucleus and cytoplasm of primary fibroblast from mice heart with TGF-β1 stimulation was performed with GAPDH as a loading control. **D** Representative immunostaining images for STAT1 (red) in primary fibroblast from mice heart with TGF-β1 stimulation. Magnification 1000×. *n* = 6, per group, *P < 0.05, ***P < 0.001, ****P < 0.0001.

Co-immunoprecipitation (Co-IP) analysis demonstrated that CARMA3 interacted with STAT1 in primary CFs isolated from WT and CARMA3 KO mice following TGF-β1 stimulation (Fig. 6A). Western blot analysis showed increased STAT1 phosphorylation in CARMA3 KO CFs under TGF-β1 stimulation (Fig. 6B). Meanwhile, the protein level of p-STAT1 in the nucleus increased in CARMA3 KO CFs following TGF-β1 stimulation (Figure S4). In addition, STAT1 protein expression was significantly higher in the nucleus and lower in the cytoplasm of CARMA3 KO CFs (Fig. 6C). Fluorescence staining further confirmed increased STAT1 expression in the nucleus of CARMA3 KO CFs (Fig. 6D)

### STAT1 inhibition improves cardiac function and inhibits fibroblast activation under pressure overload

**Fig. 7:**
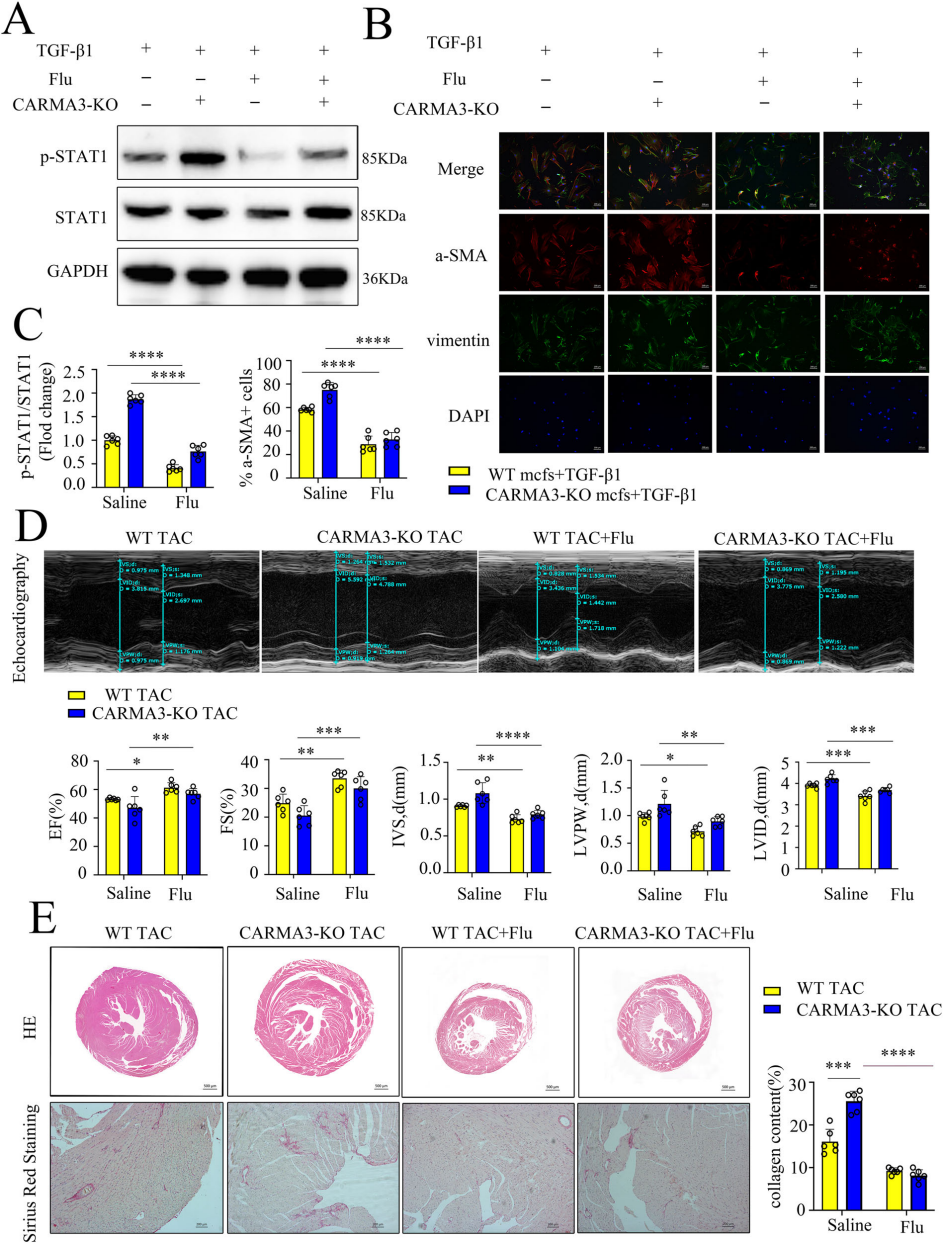
STAT1 inhibition improves cardiac function and inhibits fibroblast activation under pressure overload. **A**, **C** Western Blot of p-STAT1 and STAT1 in primary fibroblast from mice heart with or without TGF-β1 and fludarabine stimulation was performed with GAPDH as a loading control. **B**, **C**. Representative immunostaining images for α-SMA (red) and vimentin (green) in primary fibroblast from mice heart with or without TGF-β1 and fludarabine stimulation. Magnification 100×. **D** Echocardiography of wild-type and CARMA3 KO mice heart with or without fludarabine stimulation after TAC and EF, FS, IVSD, LVPW, and LVID. **E** Images of HE (Magnification 50×) and picrosirius red staining (Magnification 100×) of mice heart and quantification of fibrotic area of mice heart. *n* = 6, per group, *P < 0.05, **P < 0.01, ***P < 0.001, ****P < 0.0001.

To investigate the role of STAT1 in hypertrophic cardiomyopathy, primary CFs from WT and CARMA3 KO mice were treated with fludarabine to impede STAT1 phosphorylation. Fludarabine treatment significantly reduced STAT1 phosphorylation (Fig. 7A, C). In CARMA3 KO CFs, combined treatment with TGF-β1 and fludarabine led to a more pronounced decrease in the proportion of α-SMA-positive cells (Fig. 7B, C). Echocardiography analysis of mice subjected to TAC combined with STAT1 inhibition revealed significant improvements in cardiac function, as shown by decreased IVSD, LVPW, and LVDI, along with increased LV EF% and FS% (Fig. 7D). Histological analysis using hematoxylin and eosin (H&E) and Sirius Red staining demonstrated a more substantial reduction in myocardial hypertrophy and cardiac fibrosis in CARMA3 KO mice treated with fludarabine (Fig. 7E).

## Discussion

Myocardial hypertrophy is a pathological adaptive response of the heart to sustained pressure or volume overload, leading to adverse cardiac remodeling and myocardial fibrosis [6]. Several studies have implicated the profibrotic signaling of cardiac fibroblasts as a critical contributor to the pathological process of cardiac fibrosis [25]. Previous research has shown that the CARMA3-BCL10-MALT1 (CBM) complex, formed by CARMA3, can regulate myofibroblast activation during Ang-II-induced liver vascular inflammation [21]. Nevertheless, the role of CARMA3 in cardiac fibroblasts and its underlying molecular mechanisms remain poorly understood. Therefore, further investigation into the effects of CARMA3 on myocardial fibrosis and hypertrophy, as well as its specific actions within fibroblasts, is essential.

In the present study, we observed a downregulation of CARMA3 expression in activated human cardiac myofibroblasts, based on analysis of the public dataset GSE174490. Human primary fibroblasts were then isolated from atrial tissue obtained from cardiac surgery patients. Stimulation with TGF-β1 significantly activated these fibroblasts into myofibroblasts, accompanied by increased expression of the fibrosis marker Collagen3 and the myofibroblast markers POSTN and α-SMA. These findings were consistent with the results from GSE174490, further supporting the decrease in CARMA3 expression in myofibroblasts. We also observed a reduction in CARMA3 expression in thickened myocardial tissue as myocardial fibrosis progressed, with CARMA3 expression primarily localized to fibroblasts. Similar findings were found in murine heart tissues exhibiting myocardial hypertrophy. Previous studies have shown that pressure overload-induced chronic injury in mice post-TAC leads to congestive heart changes, resulting in chronic myocardial fibrosis, cardiac hypertrophy, and pathological alterations associated with heart failure [26, 27]. To further evaluate the impact of CARMA3 deficiency on cardiac fibrosis, we conducted a dual validation approach by establishing a cardiac hypertrophy model through Ang II stimulation.

Herein, CARMA3 gene knockout exacerbated myocardial hypertrophy and cardiac dysfunction in mice with congenital coarctation of the aorta or Ang II stimulation. In addition, CARMA3 knockout increased the mortality rate in mice post-TAC. While no deaths were observed in WT mice induced by Ang II, a significant increase in mortality was noted in Ang II-induced CARMA3 knockout mice. This increase in mortality may be due to acute heart injury caused by TAC surgery, which often leads to obstruction of the outflow tract and subsequent congestive heart failure. Conversely, Ang II stimulation was achieved through subcutaneous infusion, providing chronic stimulation to the mouse heart, which led to hypertension and myocardial hypertrophy. WT mice developed compensatory adaptive responses during this prolonged stimulation, resulting in myocardial hypertrophy and fibrosis. However, CARMA3 knockout mice displayed insufficient compensatory function, leading to a higher mortality rate due to blood vessel ruptures [24]. In conclusion, CARMA3 gene knockout in a mouse model of myocardial hypertrophy resulted in increased mortality, more severe myocardial hypertrophy, impaired cardiac function, and exacerbated cardiac fibrosis.

Upon cardiac injury, resident cardiac fibroblasts differentiate into myofibroblasts, characterized by α-SMA expression, which leads to fibrosis through the secretion of excessive extracellular collagen and matrix [28]. Our study demonstrated a significant increase in the expression of the myofibroblast marker α-SMA, the extracellular collagen markers Collagen I and Collagen III, and the extracellular matrix markers Vimentin and POSTN in cardiac tissues of CARMA3 knockout mice. Moreover, cardiac fibroblasts isolated from TAC mice revealed that CARMA3 knockout promoted the activation of cardiac fibroblasts into myofibroblasts. Therefore, CARMA3 knockout may directly promote stress overload-induced myocardial fibrosis by regulating fibroblast activation. Numerous studies have shown that TGF-β1 can drive fibroblast activation and contribute to the development of fibrosis [29, 30]. To further explore the impact of CARMA3 knockout on myofibroblasts, we isolated primary fibroblasts from murine hearts and induced their transformation into myofibroblasts using TGF-β1. We found that the absence of CARMA3 enhanced fibroblast activation into myofibroblasts, accompanied by increased secretion of pro-fibrotic factors (POSTN, α-SMA, collagen1a1, Plod2) and pro-inflammatory factors (TGF-β, IL-6, IL-1β, TNF-α). In addition, studies have shown that CARMA3 deletion promotes myofibroblast proliferation. This continued proliferation results in sustained secretion of extracellular collagen and matrix, further accelerating the progression of myocardial fibrosis [31].

Numerous studies have emphasized the strong link between fibrosis and inflammatory responses [32, 33]. Macrophages, key regulators of inflammation, interact extensively with myofibroblasts, which are crucial in the fibrotic process. Research has shown that the absence of macrophages during the inflammatory phase can disrupt the proliferation and collagen secretion of myofibroblasts, worsening fibrosis. Furthermore, a deficiency in reparative macrophages may hinder the degradation of the extracellular matrix, leading to excessive fibrosis [34]. Notably, cardiac myofibroblasts can influence the recruitment and phenotypic transformation of macrophages by releasing cytokines [35]. In our study, we stimulated primary fibroblasts from WT and CARMA3-KO mice with TGF-β1 to generate conditioned media, which was then used to culture primary macrophages from WT mice. Our results show that factors released by CARMA3-KO myofibroblasts enhanced the migratory capacity of macrophages and promoted their polarization towards the pro-inflammatory M1 phenotype while reducing polarization towards the anti-inflammatory M2 phenotype. This indicates that CARMA3 knockout can promote the ability of myofibroblasts to drive fibroinflammatory responses.

To elucidate the molecular mechanisms by which CARMA3 regulates myofibroblast activation during cardiac fibrosis and ventricular remodeling, we performed proteomic sequencing analysis on fibroblasts from the hearts of WT and CARMA3 KO mice with TAC. GO analysis revealed that the significantly differentially expressed genes were primarily localized in the extracellular region, suggesting a key role for CARMA3 knockout in modulating fibroblast responses to stimuli and regulating the immune system. These findings are consistent with our study results, demonstrating that CARMA3 knockout in fibroblasts enhances cytokine secretion, extracellular matrix production, and macrophage recruitment by myofibroblasts. Furthermore, KEGG analysis suggested the potential involvement of the JAK/STAT signaling pathway in regulating myofibroblast activation following CARMA3 knockout. Importantly, by analyzing significantly differentially expressed proteins and protein-protein interaction networks, we identified a key role for STAT1. Previous studies have shown that STAT1 is crucial in regulating the function of myofibroblasts [36, 37]. STAT1, a member of the STAT transcription factor family, is activated in response to cellular environment changes, thereby modulating target gene expression. It plays a crucial role in various biological processes, including embryonic development, organogenesis, innate and adaptive immunity, cell proliferation and differentiation, and programmed cell death [38]. Under normal conditions, STAT1 is localized in the cytoplasm; however, upon stimulation, the STAT1 pathway is activated, leading to STAT1 phosphorylation and subsequent nuclear translocation. Once in the nucleus, STAT1 regulates transcription to modulate the expression of target genes [39]. CARMA3 plays a pivotal role in mediating membrane receptor signaling and the subsequent phosphorylation of downstream molecules. Our experimental findings demonstrate that in fibroblasts stimulated by TGF-β1, CARMA3 knockout enhances STAT1 phosphorylation and nuclear translocation. Additionally, co-immunoprecipitation experiments confirmed the interaction between CARMA3 and STAT1 in cardiac fibroblasts. Studies have indicated that fludarabine can inhibit STAT1 phosphorylation and nuclear translocation [40–42]. To further investigate the role of CARMA3 knockout in promoting myocardial fibrosis, we examined its regulation of myofibroblast activation through STAT1 phosphorylation. Our findings show that inhibiting STAT1 phosphorylation with fludarabine alleviated myofibroblast activation induced by CARMA3 knockout. This intervention significantly improved cardiac function, reduced myocardial fibrosis, and mitigated ventricular remodeling in CARMA3-KO TAC mice.

One limitation of the present study is that we only demonstrated CARMA3’s regulation of the interaction between fibroblasts and macrophages. Further research should investigate the detailed mechanisms underlying this interaction. Moreover, fludarabine affects STAT1 phosphorylation throughout the body rather than specifically inhibiting STAT1 expression in fibroblasts. Thus, future studies should explore the specific mechanisms by which STAT1 functions in fibroblasts.

In conclusion, CARMA3 knockout in fibroblasts significantly enhances myofibroblast activation through the STAT1 signaling pathway, exacerbating pressure overload-induced cardiac hypertrophy and fibrosis, as well as negatively impacting cardiac remodeling and systolic dysfunction. Therefore, CARMA3 presents a promising therapeutic target for late-stage cardiac remodeling, especially in addressing myocardial fibrosis. Clinical studies involving CARMA3 inhibitors should closely monitor cardiac function, with particular attention to adverse events related to myocardial hypertrophy and fibrosis.

## Methods

Data supporting the results of this study have been made available to the journal upon request. Detailed methodological information can be found in the Supplementary Materials.

## Supplementary information

S1

S2

S3

S4

S1

S2

SUPPLEMENTAL MATERIAL

Original Data
