# Supplementary figures and images for: The role of CARMA3 in regulating fibrosis to prevent hypertrophic cardiomyopathy
Source: Cell Death Discov. 2025 Oct 6;11:429. doi: 10.1038/s41420-025-02645-z (PMC12501282; doi:10.1038/s41420-025-02645-z)

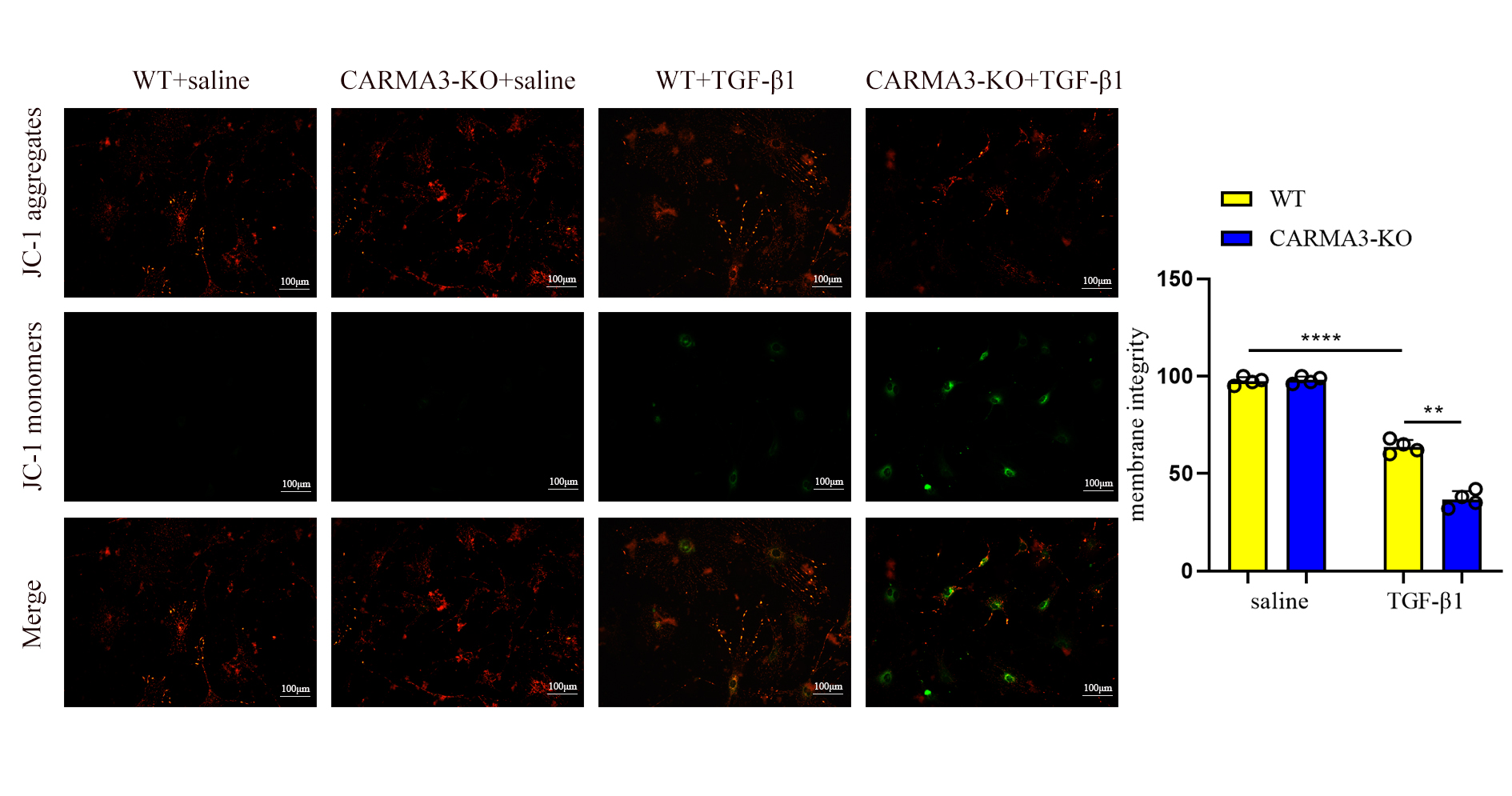

Supplement: Supplementary file 2 — S2 [file 41420_2025_2645_MOESM2_ESM.jpg]

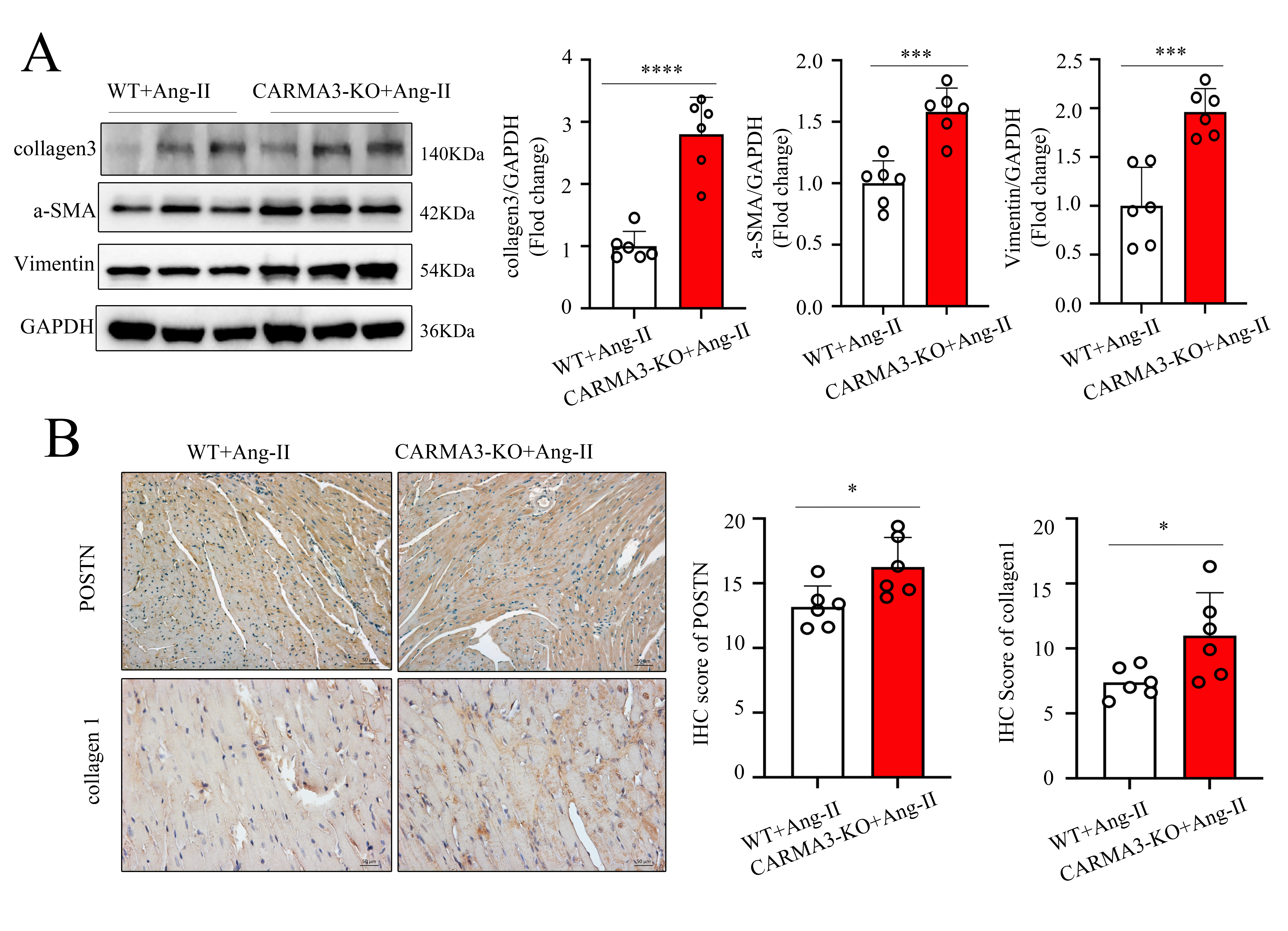

Supplement: Supplementary file 3 — S3 [file 41420_2025_2645_MOESM3_ESM.jpg]

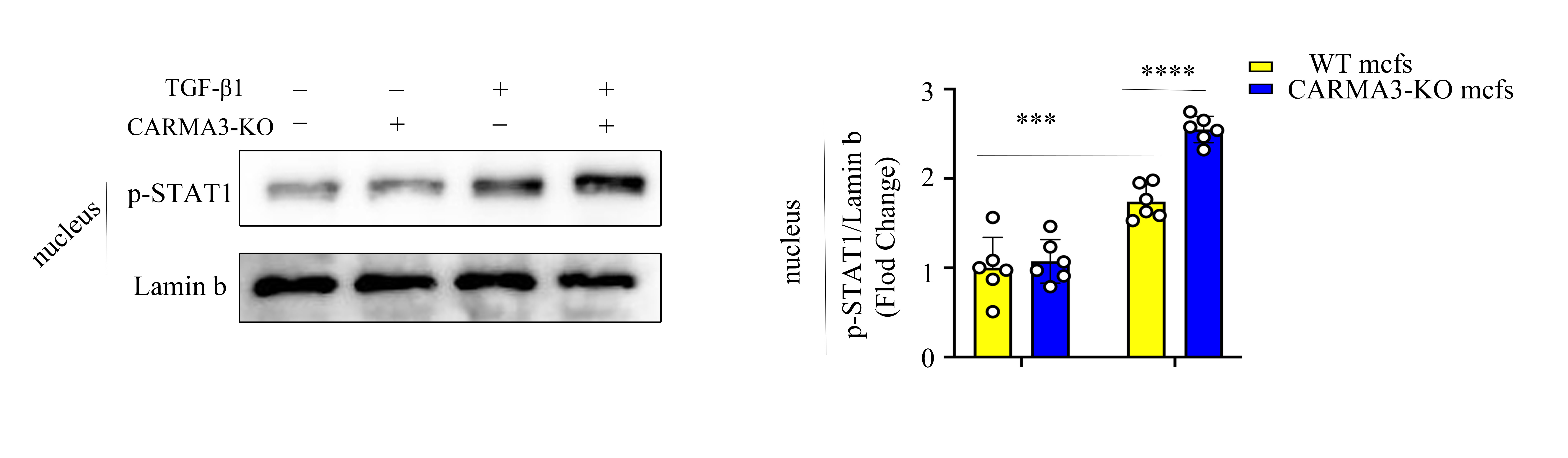

Supplement: Supplementary file 4 — S4 [file 41420_2025_2645_MOESM4_ESM.jpg]

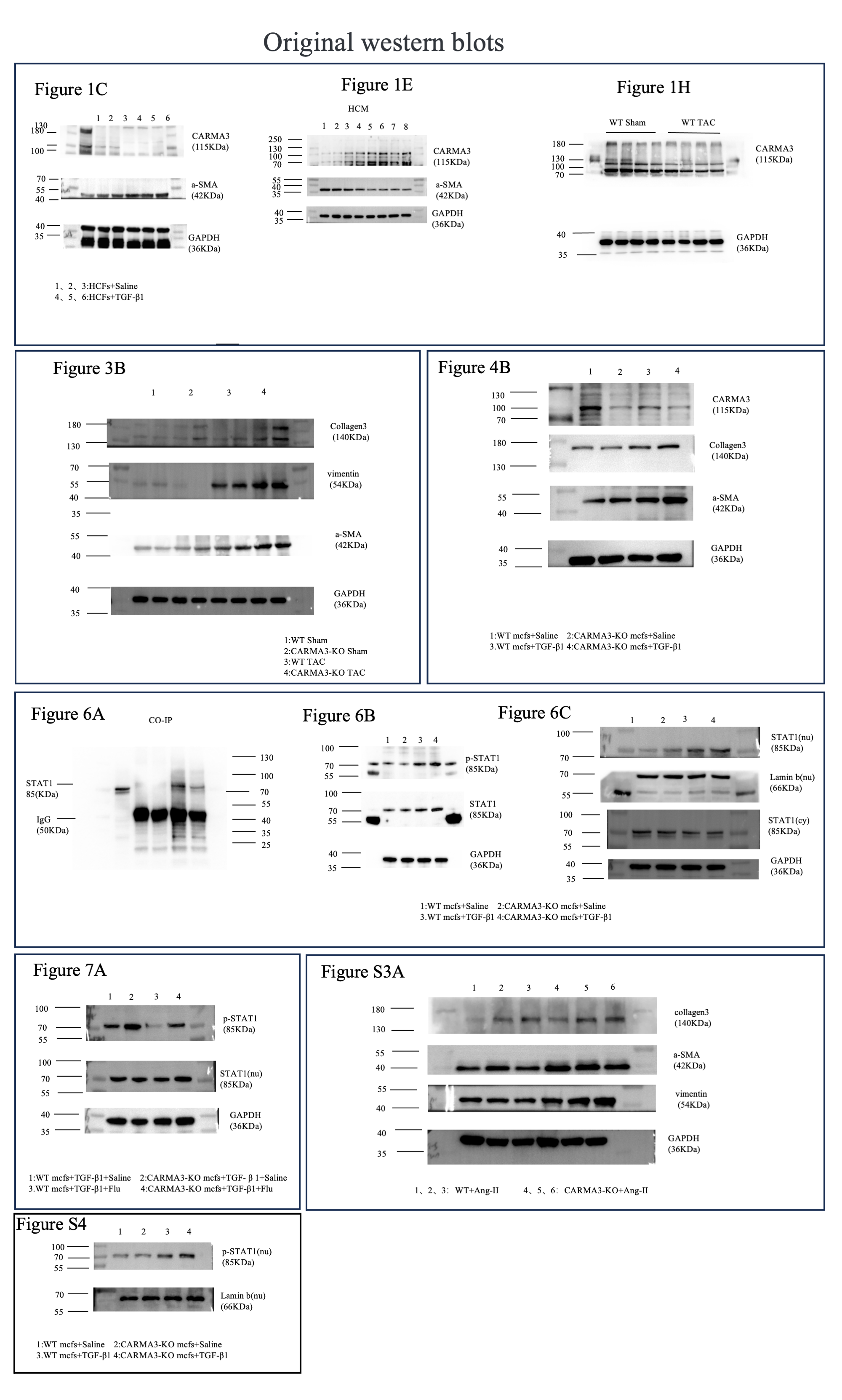

Supplement: Supplementary file 8 — Original Data [file 41420_2025_2645_MOESM8_ESM.tif]
