# Supplementary material for: The role of CARMA3 in regulating fibrosis to prevent hypertrophic cardiomyopathy
Source: Cell Death Discov. 2025 Oct 6;11:429. doi: 10.1038/s41420-025-02645-z (PMC12501282; doi:10.1038/s41420-025-02645-z)
Supplement: Supplementary file 5 — S1 [file 41420_2025_2645_MOESM5_ESM.docx]

| **Tabel S1. Characteristics of HCM patients undergoing Morrow surgery** | | | | | | | |  |  |
| --- | --- | --- | --- | --- | --- | --- | --- | --- | --- |
| patients |  | 1 | 2 | 3 | 4 | 5 | 6 | 7 | 8 |
| Age(years) | | 64 | 60 | 70 | 71 | 70 | 60 | 55 | 69 |
| Gender | | Female | Male | Male | Female | Female | Female | Female | Female |
| Height（m） | | 1.58 | 1.72 | 1.65 | 1.6 | 1.56 | 1.58 | 1.62 | 1.55 |
| Weight(Kg) | | 55 | 78 | 59 | 55 | 51 | 66 | 61 | 55 |
| Hypertension | | N | Y | Y | Y | Y | Y | N | Y |
| IVSD（mm） | | 17 | 19 | 20 | 16 | 18 | 15 | 14 | 14 |
| LVPWD（mm） | | 12 | 12 | 15 | 12 | 12 | 11 | 11 | 11 |
| LVDd(mm) | | 43 | 65 | 43 | 44 | 49 | 40 | 41 | 44 |
| SV(ml) | | 54 | 119 | 54 | 53 | 72 | 45 | 47 | 65 |
| EDV(ml) | | 157.49 | — | — | 88.41 | 160 | 84 | 130 | - |
| EF(%) | | 64 | 55 | 64 | 60 | 64 | 65 | 65 | 67 |

IVSD（Interventricular Septal Thickness at Diastole），LVPWD（left ventricular posterior wall dimensions），LVDd(Left ventricular end diastolic dimension),SV（stroke volume），EDV（Enddiastolic volume），EF（ejection fraction）
