# Supplementary material for: The role of CARMA3 in regulating fibrosis to prevent hypertrophic cardiomyopathy
Source: Cell Death Discov. 2025 Oct 6;11:429. doi: 10.1038/s41420-025-02645-z (PMC12501282; doi:10.1038/s41420-025-02645-z)
Supplement: Supplementary file 6 — S2 [file 41420_2025_2645_MOESM6_ESM.docx]

| ***Table S2: Primers used for qRT–PCR analysis*** | |  |
| --- | --- | --- |
|  | **Forward** | **Reverse** |
| POSTN | AATTAGGCTTGGCATCTGCTCT  G | CACCGTTTCTCCCTTGCTTACTC |
| ANP | ATCTGCCCTCTTGAAAAGCA | ATCTGCCCTCTTGAAAAGCA |
| BNP | ATCGGCGCAGTCAGTCGCTT | GGTGGTCCCAGAGCTGGGGAA |
| b-MHC | AGATCGAGGACCTGATGGTG | GATGCTCTTCCCAGTTGAGC |
| Col1a1 | ATGGATTCCCGTTCGAGTAC | ATGGATTCCCGTTCGAGTAC |
| fibronectin1 | GACCAGTGCCAAGATTCAGAG  ACC | TTCCTTCCAGCGACCCGTAGAG |
| Col3a1 | CCCAACCCAGAGATCCCATT | CCCAACCCAGAGATCCCATT |
| 18s | AGTCCCTGCCCTTTGTACACA | CGATCCGAGGGCCTCACTA |
| a-SMA | GTCCCAGACATCAGGGAGTAA | TCGGATACTTCAGCGTCAGGA |
| TGF-b | TGTTAAAACTGGCATCTGA | GTCTCTTAGGAAGTAGGT |
| Il-6 | ACTTCCATCCAGTTGCCTTCTTG  G | TTAAGCCTCCGACTTCTCAAGTG  G |
| IL-1β | CCAGCTTCAAATCTCACAGCAG | CTTCTTTGGGTATTGCTTGGGATC |
| Arg1 | GAACACGGCAGTGGCTTTAAC | TGCTTAGCTCTGTCTGCTTTGC |
| Egr2 | TGCTAGCCCTTTCCGTTGA | TCTTTTCCGCTGTCCTCGAT |
| TNF-a | ACGGCATGGATCTCAAAGAC | AGATAGCAAATCGGCTGACG |
| Ccl2 | CACTCACCTGCTGCTACTCATT  CG | CTTCTTTGGGACACCTGCTG |
