# Supplementary material for: The role of CARMA3 in regulating fibrosis to prevent hypertrophic cardiomyopathy
Source: Cell Death Discov. 2025 Oct 6;11:429. doi: 10.1038/s41420-025-02645-z (PMC12501282; doi:10.1038/s41420-025-02645-z)
Supplement: Supplementary file 7 — SUPPLEMENTAL MATERIAL [file 41420_2025_2645_MOESM7_ESM.docx]

**Expanded Methods and Materials**

**1.*Human heart samples***

Human myocardial tissue specimens were obtained from patients undergoing MORROW surgery at the Cardiac Surgery Center of Nanjing First Hospital. All patients provided informed consent, and all experiments involving human myocardial tissue samples were approved by the Ethics Committee of Nanjing First Hospital with the ethical approval number KY20190404-03-KS-01, following the principles of the Declaration of Helsinki. The basic characteristics of the patients are in Table s1.

**2.*Mice***

The C57BL/6 and CARMA3-KO mice were procured from the Institute of Model Animals, Nanjing University. This study was approved by the Animal Care and Use Committee of Nanjing Medical University. WT and CARMA3-KO male mice at 8-10 weeks of age and the similar weight (19-25 g) were included in the experiment, each experimental group included 15 mice. All mice were randomly divided into corresponding experimental groups. The animals were maintained under controlled conditions with an ambient humidity range of 50-60 °C and an ambient temperature range of 22-24°C for a 12-hour light-dark cycle, ensuring ad libitum access to food and water for all experimental mice.

**2.1．Hypertrophic cardiomyopathy(HCM) induced by TAC surgery**

The TAC model and sham surgery were performed by the same physician who was blinded to the treatment groups. In conclusion, the mice were anesthetized and artificially ventilated, then the transverse aorta was ligated by 6-0 nylon suture together with a blunted 27-gauge needle, which was later pulled out. After TAC, the mice with a pressure gradient ranging from 50 to 70 mmHg were used for further study. Cardiac tissue was harvested six weeks after TAC surgery.

**2.2．****Hypertrophic cardiomyopathy(HCM) induced by Ang-II**

In conclusion, to induce the hypertrophic cardiomyopathy, male mice were subcutaneously implanted with an osmotic minipump (ALZET, model 2004, ALZA Corp, Mountain View, CA) in the middle of the scapula via a small incision, delivering saline or Ang II (Sigma A9525, Sigma-Aldrich, St Louis, MO) at a rate of 1 mg/kg/min for 28 days. At the end of the injection, the mice heart tissues were harvested.

**3.** ***Echocardiography***

Echocardiography was performed using a Vevo 2100 instrument (Visual Sonics) to record the cardiac function. The mice were anesthetized with isoflurane, to keep the heart rate (HR) at >400 bpm. The echocardiographic parameters, including EF%, FS%, IVS, LVPW, LVID, LV mass and heart rate were measured from M-mode images obtained from short-axis view visualizing both papillary muscles. The pressure gradient was measured from pulse wave (PW) Doppler images obtained from short-axis view visualizing transverse aortic constriction.

**4.*****Primary fibroblast culture***

Mouse cardiac fibroblasts were prepared as follows: complete heart was minced into 1-2mm^3^ pieces and digested with cardiac tissue digestion buffer(600U/ml collagenaseII(C2-28-100MG,Sigma), DNase I 60U/ml(1121MG010, Biofrox), and 5%FBS in Hank’s buffered saline solution(C14175500BT, Gibco) at 37°C with shaking for 20-30min)). Cells suspension was then filtered through a 40-uM Cell sieve(HK2183 , BD Falcon), and suspend cells were cultured in a 0.2% gelatin-coated culture flask using DMEM supplemented with 1% P/S and 20% FBS for 2.5h and washed off other cells that were not attached. All cell culture protocols were performed at 37°C in a humidified 5% CO_2_ environment. Isolated cardiac fibroblasts (<3 passages) were seeded with a density of 15 000 cells/cm^2^ in a Costar 12-well or in a 24-well plate for immunofluorescence staining. After 48 h, fibroblasts were washed with PBS and starved overnight with DMEM medium containing 1% P/S in serum-free condition. Subsequently, the following treatment conditions were used to manipulate cardiac fibroblasts activation: recombinant mouse TGFb1 (10 ng/mL, 763102, Biolegend) and Fludarabine(100uM, S1491, Selleck).

Mouse cardiac fibroblasts were isolated from adult heart 6 weeks post-TAC. After isolated cardiac fibroblasts as described above, fibroblasts were cultured for 16 h to perform staining or qRT–PCR analysis. This method allowed us not to culture the fibroblasts for prolong period in plastic and to characterize the pro-fibrotic nature of in vivo fibroblasts.

Human primary cardiac fibroblasts were isolate from the right atrium, which obtained from patients by cardiac surgery. Specific methods are described above.

**5.*Primary BMDM culture***

Bone marrow-derived macrophages (BMDMs) were isolated from the femurs and tibiae of mice. Briefly, the cavity of femurs and tibiae containing BMDMs was flushed out with sterile cold PBS. BMDMs were then centrifuged at 1000 rpm for 10 min, and pellets were resuspended in 20 mL of macrophage medium(DMEM containing 20% L929-conditioned medium, 1% P/S, and 10% FBS). The following week, fresh macrophage medium was changed periodically for culture and pooled. To determine whether the secretome from WT+TGF-β1；CARMA3-KO+TGF-β1 fibroblasts affects macrophage migration and polarization, we treated BMDMs isolated from wild-type animal with condition media collected from cultured cardiac fibroblasts of WT, CARMA3-KO mice.

**6. *Transwell migration assay***

BMDM migration assay was performed using the Transwell system (CLS3421,Corning). BMDMs (1x10^5^ cells/well) were suspended in 100 ul of serum-free DMEM supplemented with 1% P/S and loaded into the upper chambers containing 6.5 polycarbonate membrane filter. The lower chambers were loaded with 600ul of conditioned medium from 48 h cultured cardiac fibroblasts of WT or CARMA3-KO mice. BMDMs were allowed to migrate for 12 h towards the lower chamber of the filter in the cell culture incubator. The migrated cells were then fixed and stained using an anti-CD68 antibody. Dapi was used to visualize the nuclei. For quantification, the number of CD68 positive cells was counted in each well.

**7. *Western blotting analysis***

Total protein samples were extracted from left ventricular tissue or cardiac fibroblast and 30 ug of protein separated by SDS-PAGE. Nuclear and cytoplasmic proteins were prepared from the cells using nuclear and cytoplasmic extraction reagent kits (Cayman Chemical, Ann Harbor, MI, United States) according to the manufacturer’s instructions. The proteins were transferred to polyvinylidene fluoride (PVDF) membranes (Millipore), washed third in Tris-buffered saline (TBS) with Tween diluted 1:1000 (TBST; Promega), for 10min each time, then blocked with TBST containing 5% BSA for 1 h. The membranes were incubated with the following primary antibodies in TBST with Tween plus 5% BSA overnight at 4°C: anti-CARMA3 (1:1000, Ab137383, Abcam), anti-Vimentin (1:1000, 5741, CST), anti-a-SMA(1:1000, Ab28052, Abcam), anti-POSTN (1:1000, NBP1-30042, NOVUS), anti-STAT1(1:1000, 9172, CST), anti-p-STAT1(Tyr701) (1: 1000, 9167, CST), HRP-conjugated Monoclonal Mouse Anti-glyceraldehyde-3-phosphate Dehydrogenase (GAPDH) (1:5000, HRP-60004, Proteintech) and anti-Lamin b(1:1000, 12987-1-AP, Proteintech). The next day, the PVDF membranes were washed for 10min each time with TBST three times. whereafter, the PVDF membranes were incubated with Goat Anti-Mouse IgG/HRP (1:5000, bs-0296G-HRP, Bioss) or anti-rabbit IgG, HRP-linked Antibody (1:5000, 7074P2, cell signaling technology) for 1 h at room temperature. Specific proteins were detected using an Immobilon Western chemiluminescent HRP substrate (WBKLS0500, Millipore) and captured on ChemiScope (3,300 Mini, Clinx Science Instruments). The mean gray value of each band was then semi-quantified with Chemi analysis software. All presented results are representative of at least three independent experiments.

**8. *Histology and immunohistochemistry***

Briefly, adult hearts were dissected in phosphate buffered saline (PBS) and fixed in 4% paraformaldehyde overnight at 4°C. Hearts were then washed with PBS, dehydrated in an ethanol series. Paraffin sections were prepared to perform HE, Masson’s trichrome, Sirius Red staining, and other immunostaining. For immunostaining, sections were permeabilized with 0.1% Triton-X-100 in PBS and managed for antigen retrieval using antigen unmasking solution (Vector Laboratories, USA). Endogenous peroxidase activity was blocked with 3% hydrogen peroxide (H2O2) for 10 min at room temperature (RT), which was 24–25°C. The heart sections were blocked for 1.5 h using goat serum (ZLI-9022, Beijing Zhongshan Biotechnology) to prevent nonspecific binding of the antibodies. After washing with PBS, sections were incubated with CARMA3 (1:100, Ab137383, Abcam), DDR2(1: 200, sc-81707, Santa Cruz), CARMA3(1:100, sc-271849, Santa Cruz), Vimentin(1:200, 5741, CST), a-SMA(1:200, A2547, Sigma-Aldrich), Collagen1(1:100, Ab6308, Abcam), C-TNT (1:100, ab209813, Abcam), Ki67(1:200,Ab28052, Abcam) and Caspase 3(1:200, 9662, CST) diluted in an appropriate blocking condition at 4°C overnight. Sections were washed and subsequently incubated with appropriate secondary antibody from Invitrogen (Alexa Fluor 488, Alexa Fluor 568, Alexa Fluor 647 conjugated) diluted in blocking buffer for 1.5h at RT. Dapi (for immunofluorescence) (Sigma, catalogue no. D9542) in PBS for 15min. Or, sections were washed again and developed using substrate diaminobenzidine (DAB, ZLI-9018, Beijing Zhongshan Biotechnology). After washing, sections were mounted with 100% glycerol and a staining pattern was visualized using a Leica fluorescence microscope. Or, Sections were washed and subsequently incubated with goat anti-rabbit or anti-mouse IgG (KIT-5004 and KIT-5001, MXB) for 1 h at 37°C in a humidified box. Each antibody’s signal was developed using the substrate diaminobenzidine (DAB, ZLI-9018, Beijing Zhongshan Biotechnology). The sections were counterstained with hematoxylin. and photomicrographs were taken with a Zeiss SCOPE. A1 camera. The immunohistochemistry results were analyzed based on Fromowitz semiquantitative analysis scores used to score the brown chromogen intensity (range: 0–7). The average score of each slice determined by two independent observers was used for later comparison. All histological analysis were performed blind.

**9.*Total RNA extraction and quantitative real-time PCR (q RT-PCR)***

Total RNA was extracted from the left ventricle tissues, BMDMs or cardiac fibroblasts using the TRIzol Reagent (Invitrogen, 15596-026). equal amounts of RNA (1 μg) were transformed into cDNA with the PrimScriptTM RT reagent Kit with gDNA Eraser (Takara, RR047A). Quantitative TaqMan PCR was conducted with SYBR Premix Ex TaqTM II (Takara, RR082A) by the Applied Biosystems 7,500 Real-Time PCR System. All data were normalized to 18s content and are expressed as fold increase relative to the expression level in a sham-operated control littermate mouse. Primers used for qRT–PCR analysis are listed in Table S2.

**10. *4D-Label-free quantitative proteomics***

**10.1．Flow cytometry sorting**

The hearts of WT-TAC and CARMA3KO-TAC mice were digested as described previously, and the digested suspension was centrifuged into cardiomyocytes (lower precipitate) and non-myocyte suspension (upper supernatant) at 900rpm for 1min. The non-myocyte suspension was added with PBS to constant volume of 2 ml and placed in a 5mL round-bottom tube. At least, cardiac fibroblasts were identified by CD45**^－^**CD140a**^＋^**, and data on the proportion of target cells were collected and the cell population was determined for sorting.

**10.2．****Mass spectrometry experiments**

Proteins were extracted from the sorted fibroblasts by adding appropriate amount of SDT(4%(w/v) SDS, 100mM Tris/HCl, pH7.6) lysates and subjected to SDS-PAGE. The appropriate amount of protein from each sample was digested by Filter aided proteome preparation (FASP) method, and then the peptides were desalted by C18 Cartridge, and finally the peptides were quantified (OD280). Each sample was separated using a nanoliter flow rate HPLC liquid system NanoElute. Mass spectrometry was then performed on a timsTOF Pro mass spectrometer. According to the experimental requirements, the original data obtained from mass spectrometry analysis were. d files, and the general MaxQuant software (version 1.6.14) was used for library identification and quantitative analysis. By normalizing the quantitative information of protein sets obtained from experiments, a hierarchical clustering heatmap is generated using the R package (R Version 3.4) to simultaneously classify both sample and protein expression dimensions. The protein set obtained from the experiment was annotated with GO terms and KEGG pathways using Blast2GO (version: BLASTP 2.8.0+) and KOBAS (version: KOBAS 3.0) software. The distribution of each GO classification (or KEGG pathway, or Domain) in the target protein set was compared to the overall protein set using Fisher's Exact Test method, and enrichment analysis for GO annotation or (or KEGG pathway, or Domain) annotation was performed on the target protein set. Utilize information from public databases such as IntAct (http://www.ebi.ac.uk/intact/main.xhtml) or STRING (http://string-db.org/) to identify direct and indirect interactions between target proteins, and then construct an interaction network for subsequent analysis.

**11. *CO-Immunoprecipitation***

The primary fibroblasts stimulated by saline or TGF-β1 were added with NP-40 lysate to extract cell protein, and their concentration was determined by protein BCA assay. Equal amounts of protein were taken from each of the two samples in EP tubes and diluted with NP-40. Appropriate amount of CARMA3 antibody and IgG antibody was added to the diluted Protein sample and thoroughly mixed, overnight at 4° C. On the next day, the mixed Protein A agarose beads were added to the protein sample, mixed inversely at 4°C for 4 hours, and then centrifuged at 8000 rpm for 3 minutes. Leave the precipitate and clean it three times with NP-40. Then, the sample buffer was added into the protein sample and mixed, and the metal bath at 100°C for 5 minutes, and the corresponding protein expression was detected by Western Blot.

**12. *Statistical analysis***

The results of proteomics analysis were analyzed using R language and related data packages, and other data were statistically analyzed using SPSS (version 18.0). Results were analyzed with an unpaired t-test within two groups. Comparisons among three or more groups were performed using one- way ANOVA, followed by Tukey’s post-hoc test. Differences were considered significant when the *P*-value was < 0.05. (*, P < 0.05; **, P < 0.01; ***, P < 0.001; NS, not significant).

Supplemental Figures and Figure Legends

Fig. S1. CARMA3 deficiency aggravated cardiac dysfunction with Ang II stimulation. A. Changes in mice survival in wild-type and CARMA3 KO mice with Ang II stimulation(n=15, per group). B and C. Echocardiography of wild-type and CARMA3 KO mice heart and EF, FS, IVSD, LVPW, and LVID(n=6, per group). D. Images of the whole mice heart and HE staining. (Magnification 50×. n=6, per group). E. Quantification of the heart weight/body weight ratio. F. Images of Masson (Magnification 50×) and picrosirius red staining (Magnification 200×) of mice heart and quantification of fibrotic area of mice heart. (n=6, per group). G. mRNA levels of ANP, BNP, and β-MHC of mice heart after TAC(n=6, per group). H. Quantification of the wet lung weight/body weight ratio(n=6, per group). *P<0.05, **P<0.01, ***P<0.001, ****P<0.0001.

Fig. S2. CARMA3 deficiency aggravated the mitochondrial membrane potential injury. A. Representative immunostaining images for JC-1 in primary fibroblast with TGF-β1. Magnification 100×.n=4, per group.*P<0.05, **P<0.01, ***P<0.001, ****P<0.0001.

Fig. S3. CARMA3 deficiency increased the activation of myofibroblasts with Ang II stimulation. A. Western Blot of collagen3, vimentin, and α-SMA in mice heart with Ang II stimulation was performed with GAPDH as a loading control. B. Images of collagen1 and POSTN staining of mice heart and quantification of fibrotic area of mice heart. n=6, per group,*P<0.05, ***P<0.001.

FigS4. Western Blot of p-STAT1 in the nucleus of primary fibroblast from mice heart with TGF-β1 stimulation was performed with GAPDH as a loading control.(n=6, per group, ***P<0.001, ****P<0.0001)
